# Access to medicines and out of pocket payments for primary care: Evidence from family medicine users in rural Tajikistan

**DOI:** 10.1186/1472-6963-8-109

**Published:** 2008-05-23

**Authors:** Fabrizio Tediosi, Raffael Aye, Shukufa Ibodova, Robin Thompson, Kaspar Wyss

**Affiliations:** 1 Swiss Tropical Institute, Basel, Switzerland; 2 Tajik-Swiss Health Sector Reform and Family Medicine Support Project (Project Sino), Dushanbe, Tajikistan; 3 Università Bocconi, Milan, Italy

## Abstract

**Background:**

In Tajikistan it is estimated that out of pocket payments constitute two-thirds of all health spending with high proportions of these contributions through informal payments. As a consequence, access to basic care is a major concern particularly among the most needy and vulnerable groups.

This article evaluates accessibility of prescription medicines and patient expenditures for primary care services in two rural districts of Tajikistan.

**Methods:**

901 patients aged 18 years or above who had accessed primary care facilities were interviewed, using a questionnaire based on questions regarding patient's experience of visiting the health facility. To group respondents by socio-economic status, an asset index was created using principal component analysis of the information included in the questionnaires.

**Results:**

76.7% of patients were prescribed a medicine during the visits and more than 83% of them managed to obtain it. Patients spent on average US$ 9.3 on medicines, with wide variation among socio-economic groups. Around 45% of patients paid the Family Doctor. Additionally, over 41% of patients in the highest socioeconomic quintile were referred to a specialist, while only 29% of the poorest 40%.

**Conclusion:**

This survey showed that there are financial barriers potentially inactivating utilization of basic services. These barriers can only be reduced by mobilizing more public resources to fund the health sector, providing incentives for family doctors to stop requiring payments from patients, and increasing the availability of prescription drugs in PHC facilities.

## Background

Tajikistan is one of the poorest countries of the world, with GDP per capita estimated around US$ 278 in 2005 [1,2] (PPP international US$ 1260) [3], and over 60% of the population reported living below the poverty line [4]. As other former Soviet Union countries [5], Tajkistan inherited a health system characterized by universal entitlement to comprehensive and free care, highly specialized and centralized services and an emphasis on curative in-patient care [6,7]. The bias towards in-patient care has resulted in less emphasis on primary care. Moreover reproductive health, maternal and child health, tuberculosis, HIV/AIDS control, immunization and health promotion are conceived and provided as vertically organised programmes and are separated from curative services.

Health expenditure by Government dropped from about 6% of GDP in early 1990's [8] to 1% in 2005. Planned governmental health care funding for 2006 was in the range of US$ 4 per capita. If patient and donor contributions are added, total health expenditure is around US$ 14 per year [2]. It is estimated that out of pocket payments constitute two-thirds of all health spending [8] with high proportions of these contributions through informal payments. Indeed, as state health workers are amongst the lowest paid workers in Tajikistan, with salaries often delayed and arrears of several months being common, informal payments and in-kind gifts from patients constitute the main source of income for many physicians and nurses. As a consequence, access to basic care is a major concern particularly among the most needy and vulnerable groups. In fact, the Tajik Living Standard Survey found that the poorest 20% of the population are not only more frequently sick but they also use health services less often due to financial barriers [2,9]

This article presents the methods and results of a survey to evaluate patient expenditures for primary care services and accessibility of prescription medicines in two rural districts where Family medicine has been recently implemented. The implications of the findings are discussed with conclusions for policy.

## Methods

### Setting

The survey was conducted by the Tajik-Swiss Health Sector Reform and Family Medicine Support Project (Project Sino) [10] in the districts of Dangara and Varzob. These are rural districts with a combined population close to 165,000 people. Primary Health Care (PHC) is provided in these districts by recently re-trained family doctors and nurses in 28 Rural Health Centers (RHCs), 2 ambulatories, and 54 subordinated Medical Houses (MHs). The survey was conducted as part of the activities included in a jointly signed project agreement between the Ministry of Health of Tajkikistan and the Swiss Agency for Development and Cooperation (which funded Project Sino).

### Study population and sample size

A sample of 1000 respondents was selected to represent the users of primary care facilities in the two districts. Fifteen facilities were sampled, nine in Dangara and six in Varzob balancing remote and more accessible areas and representing 50% of the PHC facilities in the two districts.

The survey included patient respondents 18 years of age or above who had accessed these facilities. The number of interviewees from the two districts was proportional to the number of adult patients that visited the facilities of each district and, within each district the number of respondents from each facility was proportional to the total number of patients at that facility in year 2004 as reported in the Health Management Information System (HMIS).

The total number of visits in all PHC facilities of the two districts was reported to be around 112,000 (0.7 visits per capita) during 2004. The names of persons who had visited the facilities were taken from the registry of the HMIS. To minimise recall bias, the most recent visitors of each facility were selected first, until the predefined number of respondents was reached. All patients included in this survey had visited a facility in April 2005 and they were interviewed at home.

### Questionnaire and interviewers

The study used a questionnaire [11] probing patients' experiences in visiting the health facility adapted to the context of Tajikistan and to the objectives of the study. The questionnaire contained specific questions relating to services used, accessibility of health services, and patient payments. Information on patient such as age, gender, level of education, and living standards was also included.

The questionnaire was administered by 26 trained interviewers who were selected from the population of the districts. The majority of them were female teachers, although in some locations male interviewers were also involved. Each interviewer interviewed approximately 30–40 patients depending on the area covered and the distances between the houses of respondents. All interviews were carried out in May and June 2005, which are not likely to be months with highest morbidity patterns.

### Data analysis

Data were analyzed in SPSS 14.0, using Pearson Chi-square tests to compare categorical data, while Kruskal Wallis Tests were used to compare means of different populations to account for non normality of distributions. In order to assess the determinants of patients' expenditures on medicines a linear regression model was run using the logarithm of expenditure in medicines as dependent variable and the following explanatory variables: age, number of years of education, number of visits in last 12 months, self assessed health status, district, asset index.

To group respondents by socio-economic status, an asset index was estimated using the information included in the questionnaire on nine variables regarding the characteristics of the household's dwelling, water, sanitation, the possession of car and television, and the consumption of meat. The asset index approach seemed to be appropriate to obtain a welfare ranking within a purely rural sample. In fact, although it has been proved possible to collect household income and expenditure data in Tajikistan [12], there are significant difficulties and pitfalls in this type of survey in developing countries [13]. The asset index used the principal component methodology of Filmer and Pritchett [14] with one continuous variable. According to the scores, and following Filmer and Pritchett approach, each subject was then assigned to the bottom 40%, the middle 40%, or the top 20% of all patients in the survey. These are then called the poorest, middle and "best off" of the sample. To check for internal coherence of the asset index the mean value of the asset variables across the poor, middle, and richest groups are shown in Table 1, and all values go in the expected direction.

**Table 1:** Asset index

					***Means***		
	
**Variables in Asset index**	**Scoring factor**	**Mean**	**Std. Deviation**	**Scoring factor/Std. Deviation**	**Poorest 40%**	**Middle 40%**	**Richest 20%**
*N persons per room*	-0.089	2.38	1.08	-0.082	2.802	2.169	1.958
*Main source of drinking water – Tap at home*	0.271	0.27	0.45	0.608	0.018	0.216	0.886
*Main source of drinking water – Public tap*	-0.222	0.23	0.42	-0.530	0.479	0.102	0
*Type of toilet – Hole*	0.381	0.97	0.18	2.151	0.921	1.000	1.000
*No toilet*	-0.341	0.02	0.14	-2.496	0.043	0	0
*Frequent meet consumption*	0.203	0.17	0.38	0.537	0.018	0.157	0.491
*Possess a car*	0.203	0.23	0.42	0.481	0.052	0.257	0.533
*Possess a TV*	0.102	0.85	0.36	0.283	0.723	0.883	1.000
*Dwelling all in good materials*	0.228	0.35	0.48	0.480	0.119	0.394	0.707

Asset index		0	0.98		-0.224	0.802	1.976

Each variable besides number of person per room takes the value 1 if true, 0 otherwise. Scoring factor is the "weight" assigned to each variable (normalized by its mean and standard deviation) in the linear combination of the variables that constitute the first principal component.

Lastly, a logistic regression model was run with the referral to a specialist (binary response: yes or no) as the dependent variable and the following explanatory variables: age, gender, number of years of education, number of visits patients had in the last months, district, self-assessed health status, asset index.

## Results

### Characteristics of patients

Out of the thousand selected patients who visited the health facilities in the two districts, 901 were interviewed at home, while 99 were not reached or refused the interview. Most of respondents were women (65%), the median age was 37 years, and the mean number of years of education was 9.8 years.

58% of subjects interviewed reported a self-assessed health status as very good, good or satisfactory, while around 41% reported to be in poor health status and only 0.7% to be in very poor health (Table 2). The respondents had, on average, 3.5 primary care visits in the last 12 months, over 27% of respondents were not diagnosed with a specific disease during the visit, 16% had a disease of the respiratory system, and 13.6% one of the digestive system, while only 5.7% were pregnancies, or conditions related to childbirth.

**Table 2:** Characteristics of patients included in the survey

	**N**	**%**
Gender		
Female	584	65.2%
Male	312	34.8%
Median age	37	
Median N year of education	10	
Self assessed health status		
Very good	66	7.3%
Good	48	5.3%
OK	409	45.5%
Poor	369	41.1%
Very poor	6	0.7%
N of visits in the last 12 months		
Mean (Std.Dev.)	3.5 (3.3)	
Median	2	

### Health care received by patients and out of pocket payments

During visits 80% of patients had their blood pressure measured, 61.5% of patients had a conversation with the attending family doctor on preventative activities related to healthy lifestyles; and slightly more that one third of patients were referred to a specialist (Table 3). Around 49% of patients had also a contact with a family nurse during the visit.

**Table 3:** Health services received by patients

	**N**	**%**
*Did the Family Doctor discuss any preventative activities with you (e.g. smoking, diet, alcohol)*		
Yes	554	61.5%
No	302	33.5%
Can't remember	40	4.4%
*Did the Family Doctor or other health worker test your blood pressure?*		
Yes	717	79.6%
No	175	19.4%
Can't remember	8	0.9%
*Did the doctor send you to a specialist?*		
Yes	310	34.4%
No	581	64.5%
Can't remember	8	0.9%
*Did the Family Doctor prescribe medicines for you during the consultation?*		
Yes	691	76.7%
No	207	23.0%
*Did you have contact with a Family Nurse during your visit?*		
Yes	435	48.9%
No	454	51.1%

The majority of patients – 76.7% – were prescribed a medicine during the visits. Additionally, more than 83% of the patients who were prescribed a medicine managed to obtain it (Table 4). Among 17% who could not obtain the medicines, the main reasons were a lack of financial resources (58.6%), absence of a pharmacy nearby (11.2%), and medicines not available in pharmacy's stock (10.3%).

**Table 4:** Access to medicines, expenditure (in US$) and payments to Family doctors

	**N**	**%**
Did you obtain the medicines prescribed by the Family Doctor?*		
Yes	572	83.3%
No	115	16.7%
If you did not obtain (all) the prescription medicines, why not?**		
No pharmacy near by	13	11.2%
No money	68	58.6%
Pharmacy did not have medicine in stock	12	10.3%
Did not feel I needed this medicine	4	3.4%
Other	19	16.4%
Did you give any money to the Family Doctor?*		
Yes	396	45.0%
No	484	55.0%

	**Mean (US$)**	**Median (US$)**
*How much money did you spend on these medicines?*	9.3	4.9
*How much money did you spend traveling to obtain these medicines?*	1.2	0.7
*How much money did you give to the Family Doctor?*	1.6	0.9
*What was the approximate value of the non monetary gifts to Family Doctor?*	0.4	0

*missing = 151;**missing = 45

Patients spent on average US$ 9.30 on medicines, while the median expenditure was US$ 4.90. Around 47% of patients reported to have spent some money for traveling to obtain the medicines and these expenditures were on average US$ 1.20 (the median was US$ 0.7). The total mean expenditure per patient to purchase medicines was US$ 10.50 (which is over 45% of the mean monthly per capita income in Tajikistan), while the median value is lower.

The medicines prescription rate was similar across socio-economic groups, while the proportion of patients who managed to obtain the medicine prescribed was higher in the highest socioeconomic quintile (93.1%) than in the lowest 40% (75.5%) and the differences were statistically significant (Table 5). There was no statistically significant difference across socio-economic groups in the reasons why some patients did not obtain the medicine prescribed, the lack of financial resources being the main one in all groups.

**Table 5:** Prescription of medicines by Asset index

	**Poorest 40%**	**Middle 40%**	**Richest 20%**
Did the Family Doctor prescribe medicines for you during the consultation?*			
Yes	78.3%	73.4%	79.0%
Did you obtain the medicines prescribed by the Family Doctor?**			
Yes	75.8%	87.0%	93.1%
If you did not obtain (all) the prescription medicines, why not?*			
No pharmacy near by	7.8%	13.9%	25.0%
No money	64.1%	52.8%	62.5%
Pharmacy did not have medicine in stock	9.4%	13.9%	-
Did not feel I needed this medicine	3.1%	5.6%	-
Other	15.6%	13.9%	12.5%

* Linear-by-Linear Association test (LLA) p > 0.05; ** LLA p < 0.05

The mean expenditure on medicines was US$ 7.70 among the poorest 40%, US$ 9.40 in the middle 40%, and around US$ 13.50 in the "best off" 20% (Table 6). These mean values hide a wide variability, with standard deviations in the order of close to two times the means, but the differences across groups are statistically significant. The mean expenditure on traveling to obtain the medicines was similar across socio-economic groups as well as the payments to family doctors (both in money and in kind). The results of the regression model showed that a higher number of visits in the last 12 months were associated with higher expenditure, as well as being in Varzob district and more educated, and being better off as estimated by the asset index (Table 7).

**Table 6:** Expenditure on medicines by Asset index (US$)

	***Poorest 40%***		***Middle 40%***		***Richest 20%***	
			
	*Mean US$*	*Median US$*	*Mean US$*	*Median US$*	*Mean US$*	*Median US$*
*How much money did you spend on these medicines?*^*a*^	7.7	3.9	9.4	5.6	13.4	6.6
*How much money did you spend traveling to obtain these medicines?*^*b*^	1.0	0.7	1.5	0.3	1.1	0
*How much money did you give to the Family Doctor?*^*c*^	1.7	1.0	1.4	0.7	1.2	0.66
*Did you give any non-monetary gifts to the Family Doctor? What was the approximate value of the gift?*^*d*^	0.3	-	0.6	-	0.2	0

a Kruskal Wallis Test = 18.412 p = ..000; b Kruskal Wallis Test = 4.910 p = .086; c Kruskal Wallis Test = 4.302 p = .116; d Kruskal Wallis Test = 9.635 p = .008

**Table 7:** Results of the regression on expenditure in medicines

***Coefficients***	***Unstandardized Coefficients***		***Standardized Coefficients***	***t***	***Sig***.
Variables*	*B*	*Std. Error*	*Beta*		
(Constant)	2.169	0.083		26.044	0.000
N of visits in last 12 months	0.060	0.016	0.151	3.748	0.000
**Asset index**	0.195	0.055	0.145	3.549	0.000
Rayon (Varzob)	0.787	0.115	0.278	6.826	0.000

R = 0.372; R^2 ^= 0.138; Adjusted R^2 ^= 0.134

* only statistically significant variables are included in the table

Around 45% of patients reported to have paid the family doctor, and among those who paid the mean payment was US$ 1.60 while the median was US$ 0.90, plus another average value of US$ 0.40 for non monetary gifts (Table 4).

**Figure 1:**
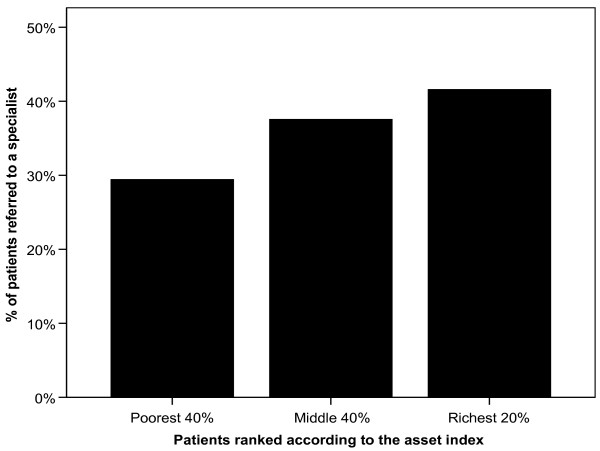
Percentage of patients referred to a specialist – by socio economic group.

Interestingly, over 41% of patients in the highest socioeconomic quintile (top 20%), ranked according to the asset index, were referred to a specialist, while only 37.4% of those in the middle 40% and 29% of the poorest 40% (Figure 1). In order to further explore this issue a logistic regression model was run with the referral to a specialist as the dependent variable. The results show that living in Dangara district led to an increased probability of being referred to a specialist as well as being better off, and having had more visits in the last 12 months (Table 8).

**Table 8:** Logistic regression showing variables influencing the odds of being referred to a specialist during the visits at the RHC

**Explanatory variable**	**Exp(B) (95.0% C.I.)**	**Sig.**
Age	1.001 (0.992–1.011)	0.694
Gender		
Female	1.00	
Male	1.072 (0.747–1.537)	0.769
N of years of education	1.044 (0.984–1.109)	0.151
**N of visits in last 12 months**	**1.063 (1.013–1.116)**	**0.012**
Rayon		
Varzob	1.00	
**Dangara**	**1.621 (1.146–2.294)**	**0.006**
Self-assessed health status		
Poor or very poor	1.00	
Very good/Good/OK	0.693 (0.407–1.180)	0.178
**Asset index**	**1.467(1.222–1.760)**	**0.000**

## Discussion

In Tajikistan only limited resources are available for the health sector and the health system is still highly focused on curative in-patient care. In fact, one of the main problems of the Tajik health system is the low level of access to primary care services which is related to both the often quite low quality of these services and to financial barriers due to endemic informal payments to health workers and the lack of public financial resources for medicine supply.

The Government of Tajikistan is implementing a health sector reform to improve access to basic services, including re-training primary care physicians as family doctors, gradually increasing the resources allocated to PHC, and changing the financing mechanisms. Dangara and Varzob districts have been piloting this reform in the recent years. This survey assessed the health care received by users of family medicines in these districts, their payments to access these services, and the potential inequalities in access among different socio-economic groups.

Interestingly in this survey the drug prescription rate was higher than that reported in Tajikistan by other surveys [8,9], and most patients managed to obtain the prescribed medicines. However there was no information on the quality of the medicines obtained by patients and on how they obtained these medicines. It is surprising that so many patients managed to obtain the medicines as in these districts the public budget for prescription medicines was very low (in 2005 it was around US$ 0,20 per capita or less than 9% of the total health budget) [15]. When this survey was carried out in the district of Dangara there were only two pharmacies (one public and one private) while in Varzob there was one (private), and only 47% of patients reported to have incurred costs traveling to obtain the medicines they were prescribed. There is anecdotal evidence that many doctors in Tajikistan often sell medicines illegally to patients. This could be an explanation for the results of this survey and it would require further evaluation because if this would be the case, it would have important implications for designing policies to increase the availability of medicines free of charge in PHC services. The fact that most patients managed to obtain the medicines they were prescribed indicates, in any case, an improvement in the availability of medicines.

Although most patients obtained the medicines they were prescribed, those better off were still more likely to obtain them than the others. Additionally the main reasons for not obtaining the medicines were lack of financial resources, the absence of a pharmacy nearby, and the absence of the medicine in the pharmacy. Unfortunately, it was not possible to further assess the doctor prescribing behavior as no information was available on clinical appropriateness of the prescriptions. The relationship between patients' expenditure, quality of care, and Family Doctors' prescription behavior should therefore be further explored.

Almost half of patients paid the Family Doctors informally. This finding, despite similar results of previous surveys in Tajikistan [8,9], confirms that changing doctor's behavior is difficult and requires an appropriate set of incentives to stop them requesting patients' payments for services that should be free.

Even in districts piloting the health sector reform there remain financial barriers to access basic services. In fact, if we add the mean informal payment to the doctor of around US$ 1.20 to the mean payment for medicines (US$ 9.30), the mean payment for a PHC visit is close to 14 times the mean daily income per capita in Tajikistan.

The financial barrier to access medicines is causing wide inequalities in the capacity to purchase high quality medicines as shown by the fact that patients in the highest socio-economic group spent on the prescribed medicines on average 74% more that those in the poorest one. This is probably due to the ability of better off patients to purchase higher quality medicines. In Tajikistan the quality of a large proportion of the medicines available in the private market is poor. The real access to medicines for poor people is likely to be much lower than the results of this survey may suggest.

The difference in the percentage of patients who were referred to a specialist among the socio-economic groups, with the richest groups being referred more often, is remarkable. This difference could be due to a Family Doctors tendency to refer to specialists more often patients who can afford to pay for specialist care. In fact, it is well known that although the whole population should be entitled to free specialist care, patients normally must pay doctors informally to access these services.

## Conclusion

Tajikistan's health care system needs major reforms and increased resources to improve the quality of services, to rationalize the delivery structure and to increase equity.

This survey showed that there are financial barriers potentially inactivating utilization of basic services, hindering access to medicines, and therefore fostering inequalities in access to basic care. These financial barriers can only be reduced by mobilizing more governmental resources from national and decentralisied entities to fund the health sector, particularly for primary care services, providing incentives for family doctors to stop requiring payments from patients, and increasing the availability of prescription drugs in PHC facilities.

The ongoing health financing reforms – the implementation of capitation payments for PHC and the introduction of formal co-payments for health services not included in a Basic Benefit Package – might be effective in reducing inequalities in access although the impact must be monitored as the danger is that formal fees add to the burden of payments. Given the tight budget constraint Tajikistan is facing and the low capacity in the country to implement reforms achievements could only be possible with long-term assistance of development partners.

## Competing interests

The authors declare that they have no competing interests.

## Authors' contributions

FT designed and conducted the analysis and led the writing up of the manuscript. RA participated in data collection and analysis. SI participated in data collection. RT contributed to the conception and design of the study and in the writing up of the manuscript. KW contributed to the conception of the study and in the writing up of the manuscript.

## Pre-publication history

The pre-publication history for this paper can be accessed here:


